# Differential Entrainment of Neuroelectric Delta Oscillations in Developmental Dyslexia

**DOI:** 10.1371/journal.pone.0076608

**Published:** 2013-10-18

**Authors:** Fruzsina Soltész, Denes Szűcs, Victoria Leong, Sonia White, Usha Goswami

**Affiliations:** Centre for Neuroscience in Education, University of Cambridge, Cambridge, United Kingdom; ARC Centre of Excellence in Cognition and its Disorders (CCD), Australia

## Abstract

Oscillatory entrainment to the speech signal is important for language processing, but has not yet been studied in developmental disorders of language. Developmental dyslexia, a difficulty in acquiring efficient reading skills linked to difficulties with phonology (the sound structure of language), has been associated with behavioural entrainment deficits. It has been proposed that the phonological ‘deficit’ that characterises dyslexia across languages is related to impaired auditory entrainment to speech at lower frequencies via neuroelectric oscillations (<10 Hz, ‘temporal sampling theory’). Impaired entrainment to temporal modulations at lower frequencies would affect the recovery of the prosodic and syllabic structure of speech. Here we investigated event-related oscillatory EEG activity and contingent negative variation (CNV) to auditory rhythmic tone streams delivered at frequencies within the delta band (2 Hz, 1.5 Hz), relevant to sampling stressed syllables in speech. Given prior behavioural entrainment findings at these rates, we predicted functionally atypical entrainment of delta oscillations in dyslexia. Participants performed a rhythmic expectancy task, detecting occasional white noise targets interspersed with tones occurring regularly at rates of 2 Hz or 1.5 Hz. Both groups showed significant entrainment of delta oscillations to the rhythmic stimulus stream, however the strength of inter-trial delta phase coherence (ITC, ‘phase locking’) and the CNV were both significantly weaker in dyslexics, suggestive of weaker entrainment and less preparatory brain activity. Both ITC strength and CNV amplitude were significantly related to individual differences in language processing and reading. Additionally, the instantaneous phase of prestimulus delta oscillation predicted behavioural responding (response time) for control participants only.

## Introduction

Children with developmental dyslexia have difficulty in the accurate neural representation of phonological aspects of speech, across languages [1]. For example, they may be poor at making decisions about whether words rhyme with each other (“cat” “hat”), or at counting syllables in words (“caterpillar”, 4 syllables). Children and adults with dyslexia also show rhythmic entrainment difficulties [2], [3]. Dyslexics are significantly more erratic at keeping time with an external isochronous rhythm (tapping to the beat) within the delta frequency range (1.5 Hz, 2 Hz, 2.5 Hz) compared to controls, and individual differences in rhythmic entrainment are related to individual differences in reading development. Further, musical training aimed at improving rhythmic entrainment in children also improves phonology and reading. Following musical training, individual differences in improvements in rhythmic accuracy show a significant relationship to individual differences in improvement in reading [4]. Difficulties in behavioural rhythmic entrainment in dyslexia are related to auditory impairments in perceiving amplitude envelope rise time, the time taken for a sound envelope to reach its highest amplitude (intensity). Children with dyslexia in English, Spanish, Chinese, Finnish, French, Hungarian and Dutch are less sensitive to the rise times of non-speech tones than children without reading impairments [5], [6]. Rise time determines rhythm in speech and also in music (rise time is the attack time of musical notes). Rise time discrimination deficits are also related to the phonological problems that characterise dyslexia across languages, at both the prosodic and sub-lexical levels [5].

Recently, a “temporal sampling” theory of developmental dyslexia has been proposed to explain the behavioural relationships between rise time discrimination, rhythmic performance and phonological difficulties [5]. The temporal sampling framework proposes that the phonological deficits found in developmental dyslexia across languages may arise in part because of atypical “temporal sampling” of the speech signal by neuroelectric oscillations. Specifically, temporal sampling theory proposes that a key impairment in dyslexia involves atypical auditory oscillatory phase-locking to slower temporal modulations below 10 Hz (see also [7]). Therefore, the temporal sampling perspective suggests that the neural origins of the phonological deficit in dyslexia are likely to involve acoustic processes that affect the efficient recovery of *syllabic structure* from the speech signal, including suprasegmental and *prosodic* structure. Processing the prosodic structure of speech depends in part on locating the stressed syllables, which carry speech rhythm. Accordingly, entrainment to the slower amplitude modulations in speech (focused on AM rates around 2 Hz and 5 Hz, the stressed syllable and syllable rates respectively) is critical for rhythmic perception and rhythmic synchronisation [7]. However, neuronal oscillatory entrainment in dyslexia in a rhythmic task has yet to be studied.

Human speech can be considered a quasi-rhythmic stimulus [8]. While speech is not perfectly periodic, the temporal aspects of syllable production are constrained within certain physiological norms. The syllables in speech are not random stochastic events (like raindrops): Rather, regularities in articulatory patterning exist that constrain the way syllables unfold in time, irrespective of speaking rate [9]. For example, in English, syllables typically occur on average every 200 ms (5 Hz), with stressed syllables (which anchor the perception of linguistic rhythm) occurring on average every 500 ms (2 Hz [5], [10]). Sensitivity to these quasi-periodic syllable and stressed syllable cues is known to be impaired in individuals with dyslexia. For example, both children and adults with dyslexia have difficulties in recognizing syllable stress [11], [12], [13], [14] (identifying strong versus weak syllables). Studies of 2- and 3-year-olds at family risk for dyslexia have indicated speech timing difficulties in children who later present with reading difficulties, suggesting that difficulties are found in speech production as well as speech perception [15], although motor production difficulties as distinct from timing difficulties are a potential confound for the production data. Children who later turned out to have dyslexia produced significantly fewer syllables per second in early childhood (4.8 at age 3 compared to 7.1 for non-risk children) and paused for longer between articulations, suggestive of early syllable-level deficits. Syllable awareness is primary in children’s phonological development across languages, as phonological awareness (the ability to recognise and manipulate sound units in words) follows a developmental sequence from syllable to onset-rime to (once reading is taught) phoneme [1]. Phonemes are the smallest sound elements in words, as represented by the alphabet. Once they learn to read, children with developmental dyslexia also show impaired abilities in phoneme-level tasks, for example phoneme deletion tasks (e.g., “say ‘bice’ without the/b/”). However, in transparent orthographies such as German, even children with dyslexia will acquire good phoneme-level skills by the age of around 10 years [16]. Phonologically, therefore, dyslexia is characterised by the inefficient development of the entire phonological system (encompassing the accurate specification of prosodic phonology, as well as the phonological structure of multi- and single-syllable words, efficient phonological memory, and efficient and rapid output of automatized phonological information such as over-learned colour names – “rapid automatized naming” or RAN [17]).

Meanwhile, recent work in auditory neuroscience has demonstrated that accurate perception of the speech signal at multiple temporal scales is important for the efficient extraction of meaningful phonological elements, and that oscillatory entrainment mechanisms may contribute to this process [18], [19], [20], [21]. It is suggested that perception of temporal modulations at slower rates (below 10 Hz, e.g., corresponding to cortical oscillations at the delta 0.5–4 Hz and theta 4–8 Hz frequency ranges) is relevant to the extraction of syllable-level and prosodic information, while perception of temporal modulations at more rapid rates (corresponding to brain activity at the gamma rate, 30–80 Hz) is relevant to the extraction of phonetic information. In oscillatory entrainment, attended inputs are assumed to align with the excitable phase of the oscillatory cycle, while unattended inputs align with the inhibitory phase [22]. Exploration of oscillatory entrainment in humans using EEG has so far mainly relied on measuring phase alignment to *rhythmic* stimulus streams [23], [24] although see also [25], [26]. Rhythmic streams were chosen partly because previous EEG work with animal models used rhythmic stimulus streams [22], [27], and also because many biological inputs occur in rhythmic and predictable patterns. Therefore, theoretically it is assumed that neuroelectric oscillations are relevant to encoding these rhythmic patterns [19], [28], [29], [30]. Phase/amplitude cross-frequency coupling mechanisms then coordinate the neural activity on multiple time scales, which for speech includes auditory and visual cross-modal phase modulation [31].

Regarding rhythmic patterning in human language, it has long been recognised that speech rhythm provides an important structural cue for infants who are acquiring their native phonology [32]. Infant studies demonstrated that neonates can distinguish languages from different rhythm classes, and since the amniotic fluid acts like a low-pass filter, this might be partly because the slower temporal modulations in speech (<10 Hz) are also audible to the foetus. Amplitude modulations at 3 Hz and 6 Hz elicit a sustained desynchronization in the theta range (4–8 Hz) in 6-month-old infants, suggestive of efficient oscillatory auditory entrainment to syllable-rate information [33]. Efficient temporal sampling of speech input via neuroelectric oscillations in the delta range could in effect provide a rhythmic framework for attention, increasing the efficiency of prediction of the next critical stimulus (the next stressed syllable [7], [34]), so that attention can be shifted effectively and behaviour can be optimised. Similar theoretical frameworks have been proposed by oscillatory cognitive models of attention and musical beat processing [35], [36]. Oscillatory phase alignment at lower frequencies has also been linked to a broadband EEG component called contingent negative variation [37], a negative deflection preceding target stimuli. The CNV reflects anticipatory attention and motor preparation in expectation of the upcoming stimulus, and is driven by top-down control from the prefrontal cortical areas [38]. Preparatory brain activity preceding stimuli is critical for efficient and succesful behaviour when stimuli are predictable, as preparatory activity pre-activates the networks necessary for fast and efficient responding. The CNV is the most commonly studied EEG marker of this preparatory activity, and has been related to attention, predictive timing and response preparation [39], [40]. In animal oscillatory work using rhythmic stimulus streams [22], [27], *delta band oscillations* appear to be a critical mechanism for attentional processing, with delta entrainment amplifying task-relevant sensory streams. In EEG work with humans, rhythmic attending has been shown to be related to dynamic modulation of delta oscillation *phase* [23]. The phase of the delta band oscillation just before a stimulus occurred predicted reaction time in a button press task, indicating that prestimulus delta phase exerted an independent effect on behaviour in neurotypical adults.

Oscillatory entrainment in EEG in attended rhythmic tasks and the CNV have yet to be studied in dyslexic adults, and we present the first such study here. Given the behavioural evidence that (a) dyslexics show poorer rhythmic entrainment to metronome beats presented at rates around 2 Hz, (b) that individual differences in entrainment at delta rates are related to individual differences in phonological development and reading, and (c) that training children using rhythmic musical games leads to improvements in tapping to the beat, phonology and reading, we expected weaker delta oscillatory entrainment in dyslexia, with a significantly reduced CNV (which would indicate less preparatory brain activity in expectation of the next “beat”). Phase locking strength and CNV amplitude were expected to be related to individual differences in phonological processing and reading. Further, in neurotypical adults, faster reaction times in rhythmic tasks are related to the rising slope of the delta oscillation [23]. If this relationship is absent in dyslexia, it could suggest *functional* differences in oscillatory processes between dyslexics and controls related to rhythmic behaviour.

Although there are no prior studies of these functional relationships, there are two prior MEG studies of neuronal oscillation and phase locking in adults with developmental dyslexia, and one prior EEG study of oscillatory phase entrainment and reading development in children using a rhythmic task. Regarding adults, Hämäläinen and colleagues [41] used MEG to present amplitude-modulated white noise at 4 temporal rates to adults with and without dyslexia in an unattended paradigm (participants watched a silent movie). Rates of 2 Hz, 4 Hz, 10 Hz and 20 Hz were presented. Consistent with the predictions of the temporal sampling framework [5], the participants with dyslexia showed significantly reduced phase locking at 2 Hz in a superior source in the right hemisphere. They also showed significantly less phase locking overall in the right hemisphere, which preferentially processes lower-frequency temporal modulations [42], [43]. In a post-hoc analysis, Hämäläinen and colleagues also found significantly *enhanced* phase locking in the left hemisphere in dyslexics in the 10 Hz condition, suggesting atypical lateralized effects at faster temporal rates also. The temporal sampling framework suggests that phase locking to faster temporal rates (e.g. gamma) will be affected by atypical phase locking at lower frequencies, for example faster oscillations may be over-weighted in dyslexia in the final speech percept, leading to the overspecification of phonetic information (so that, as in young infants, all phoneme boundaries in human languages are maintained, without specialization for processing native speech contrasts; [44], [45]).

Also taking an oscillatory perspective, Giraud and her colleagues have proposed that there could be ‘over-sampling’ at the gamma rate in developmental dyslexia, with consequences for efficient phoneme representations [43], [46]. An MEG study of gamma oscillations in dyslexia measured the auditory steady-state response (ASSR) to a complex white noise stimulus that increased linearly in modulation rate between 10–80 Hz [45]. The ASSR indicates a power increase at the rate of stimulation, which could indicate entrainment, as large networks of neurons could be aligning their excitability fluctuations to the amplitude modulation rate of the stimulus. Lehongre and colleagues [46] reported that the ASSR at 30 Hz (low gamma rate) in auditory cortex was not left-dominant in dyslexic adults, in contrast to control participants. The reduced leftward bias was correlated with measures of phonemic processing. Therefore, gamma entrainment may also be atypical in developmental dyslexia.

Regarding typically-developing children, Power and colleagues [47] recently reported a study of oscillatory entrainment to rhythmically-presented speech using a modification of the non-speech paradigm used in the current study. Children aged 13 years listened to a human voice saying the syllable “ba” repeatedly at a rate of 2 Hz. Occasionally the syllable was out of rhythm (slightly late), and children had to press a button when they detected the arrhythmic stimulus. Significant oscillatory entrainment to rhythmic speech at both the stimulation rate (2 Hz) and at the theta rate was found [47]. The theta rate is given theoretical importance for syllabic processing by Poeppel and his colleagues [19]. Individual differences in auditory theta entrainment were related to individual differences in reading development, with the children who showed stronger inter-trial phase coherence also showing better single word reading. Nevertheless, to date there is no information regarding the relationship between oscillatory phase and processing efficiency in developmental dyslexia. We present the first such study here.

## Materials and Methods

### Participants

Via the Disability Resource Centre at the University of Cambridge, we recruited 16 adults with a childhood history of dyslexia (mean age 25.8 years, 8 males, 12 right-handed), who were still documented as impaired relative to their peers and hence qualified for disability support such as extra time in examinations. We also recruited 16 controls (mean age 27.7 years; 5 males, 14 right-handed). The adults with dyslexia all showed significant literacy and phonological deficits compared to the control adults according to our own test battery, nevertheless all participants were attending university and therefore should be considered compensated dyslexics. All participants had no diagnosed visual or hearing difficulties, nor additional learning difficulties (e.g. dyspraxia, ADHD, autistic spectrum disorder, speech and language impairments) and spoke English as a first language. Five participants’ data were omitted from the analysis due to a high ratio of EEG artefacts; we retained data from 14 controls (mean age 27.5 years, SD 5.5; 4 males) and 13 dyslexics (mean age 25.8 years, SD 6.9; 8 males). Participant details for these 27 adults are shown in Table 1.

**Table 1:** Participant details.

Group	Dyslexic (n = 13)	Controls (n = 14)	One-Way ANOVA F(1,25)	Eta-squared
Chronological age in years	25.8 (6.9) Range = 17.5–41.6	27.5 (5.5) Range = 20.8–38.5	.505	0.02
Verbal IQ (WASI vocabulary subscale T score, mean = 50)	60.2 (13.1)	65.7 (6.3)	1.97	0.07
Non-verbal IQ (WASI block design subscale T score, mean = 50)	58.5 (7.8)	62.8 (6.4)	2.39	0.09
Reading standard score (WRAT)	99.3 (14.7)	115.1 (4.0)	15.0**	0.37
Spelling standard score (WRAT)	98.1 (11.6)	115.7 (6.2)	24.9***	0.50
Spoonerisms^a^	14.6 (5.2)	18.0 (2.1)	b4.57*	0.17
RAN time in seconds	36.8 (6.2)	29.6 (4.2)	b11.3**	0.34
Digit span (WAIS-R subscale score, out of 16)	9.8 (3.3)	12.6 (1.6)	8.30**	0.25
Syllable Stress: Same Word (d′)	4.1 (0.9)	4.7 (0.3)	7.17*	0.22
Syllable Stress: Different Words (d′)	1.0 (0.9)	3.3 (1.3)	28.7***	0.53

Note. Standard deviations in parentheses.

a Score out of 20;

b Degrees of freedom are (1,22).

* p<.05,

** p<.01,

*** p<0.01.

### Ethics Declaration

All participants gave written informed consent in accordance with the Declaration of Helsinki, and the study and consent procedure were approved by the Psychology Research Ethics Committee of the University of Cambridge.

### Behavioral Assessments

#### Standardized assessments

All participants were given 2 subscales of the Wechsler Abbreviated Scale of Intelligence [48], a nonverbal subscale (Block Design) and a verbal subscale (Vocabulary). Literacy skills were assessed using the untimed Wide Range Achievement Test [49] (Reading and Spelling scales). A measure of short-term memory, the Weschler Adult Intelligence Scale- Revised forward digit span subtest was also administered [50].

#### Phonology/auditory processing

Phonology/Auditory processing was measured using two tasks: Spoonerisms and Sensitivity to Syllable Stress. The Spoonerisms task was drawn from the Phonological Assessment Battery [51] (PhAB). Participants heard 10 pairs of words presented orally by the experimenter. Participants were asked to swap the onset phonemes of the pair of words (e.g. for “*sad cat*”; subject responded “*cad sat*”). Scores on this measure were out of a possible 20 points. Sensitivity to Syllable Stress was tested as follows. Two same-different judgement tasks based on 4-syllable words were administered. In the first task (Same Word), the same word was given twice, with same or different stress (e.g., *DIfficulty* and *diFFIculty;* ‘different’ judgement required). In the second task (Different Words), two different words were presented, which shared either the same (e.g., *DIfficulty-VOluntary*) or a different (e.g., *DIfficulty-riDIculous*) stress pattern. Each task comprised 80 same-different judgements. Sensitivity to syllable stress (d′) was computed for each task. Further task details can be found in Leong and colleagues’ report [14].

#### RAN (Rapid Automatized Naming)

Two versions of an object RAN task designed originally for children were administered, one based on pictures of objects whose names resided in dense phonological neighbourhoods (RAN Dense: *Cat, Shell, Knob, Thumb, Zip*), and one based on pictures of objects whose names resided in sparse phonological neighbourhoods (RAN Sparse: *Web, Dog, Fish, Cup, Book*). Participants were shown a sheet of paper with the same pictures repeated 50 times. In each case, they were asked to produce the names as quickly and accurately as possible. Performance was timed, and the two tasks were combined to give an average RAN score in seconds.

### Rhythmic Entrainment Task

The task comprised listening to a continuous rhythmic stream of 500 Hz tones of 200 ms duration with a rise and fall time of 20 ms, presented through piezoelectric insert earphones. Stimulus blocks alternated between two different isochronous rates, 2 Hz (500 ms ISI) and 1.5 Hz (660 ms ISI). We chose to use two temporal rates in order to study whether oscillatory (re)entrainment to a change in rate was slower in participants with dyslexia. However, as there was excessive blinking each time the rate changed, this was not possible. Fifteen percent of the sounds were white noise (target sounds). Participants pressed a button when they heard a target. There were 16 alternating blocks of 2 Hz and 1.5 Hz sounds, and the length of blocks was randomly varied between 80 and 120 sounds/block. In total, there were approximately 800 stimuli at each temporal rate. The task took approximately 40 minutes to complete. The normality of the RT distribution in each group was tested by the Lillefors test. Group RT distributions were compared using the non-parametric Mann-Whitney U test.

### EEG Procedure

#### EEG preprocessing

EEG data were recorded and digitised with a 24-bit A/D converter using the 129-channel EGI Geodesic Sensor Net system. The sampling rate was 500 Hz. Data pre-processing was done using Matlab. The data was recorded from DC to the Nyquist (half the sampling rate) frequency. The data was highpass filtered at 0.01 Hz and lowpass filtered at 50 Hz offline, using a zero phase-shift two-directional second order Butterworth filter. Use of a two-directional filter cancels out phase shifts due to the filtering process. Line noise (45–55 Hz) was also bandstop filtered with a third-order two-directional (non-phase shift) Butterworth filter. Epochs from −800 to 800 ms (stimulus at 0 ms) were extracted for artefact rejection. Data was re-referenced from electrode Cz to average reference. Epochs containing data points over or below ±100 µV were marked for rejection. Electrodes showing stationary and non-movement related noise across the experiment were interpolated (maximum 5 electrodes in one subject). Fifty-six percent of trials were kept for analysis (on average 270; trials per subject; s.d. 67.5, min: 190, max: 429). The relatively low ratio of retained trials (56%) was due to the passive nature of the paradigm. Since participants were not required to respond 85% of the time, they tended to move and blink relatively frequently, resulting in several rejected epochs.

#### Time-domain Event-Related Potential (ERP) analysis (CNV)

**Figure 1:**
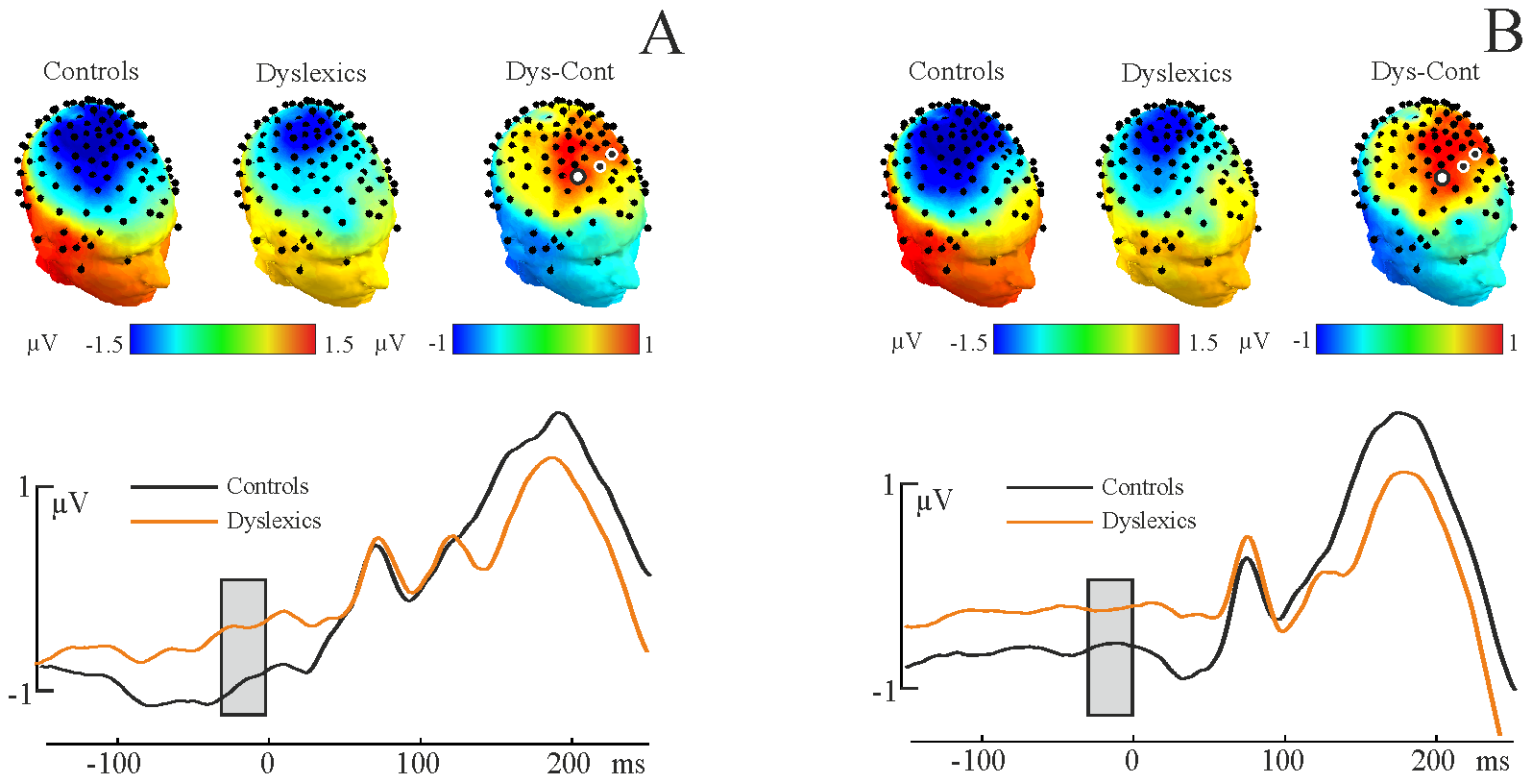
Time-domain event-related potentials by condition. Panel A: 2 Hz condition. Panel B: 1.5 Hz condition. Topographic headplots show the contingent negative variation (CNV) for both groups and the difference between the groups. Electrode showing significant amplitude difference in the prestimulus −50 to 0 ms interval after correcting for false positives are marked with larger disk. ERP traces are shown from the most significant electrode (marked with larger disk).

For the investigation of contingent negative variation (CNV) in the EEG, epochs retained after artefact rejection were baseline-corrected to the average of the epoch (−800 to 800 ms). Although the average baseline chosen here could allow post-stimulus effects to cause artifactual pre-stimulus effects, it was necessary because of the very short epochs (500 ms and 660 ms), which meant that pre-and post-stimulus intervals overlapped from trial to trial. Ideally, for the analysis of CNV, the baseline should be taken from a time interval preceding the pre-stimulus interval, but in the present case that was not possible. Consequently, the analysis of the CNV took this into account (as detailed below). A point-by-point between-group t-test was then applied to compare the CNV between groups in both conditions (2 Hz, 1.5 Hz), before each stimulus was presented (−50 to 0 ms) on three fronto-central electrodes where the CNV effect was visible (Figure 1). The p-values of multiple point-by-point t-tests were corrected for false discovery rate (FDR) utilizing the method described by Benjamini and Yekutieli [52]. The FDR correction has been shown to be an effective practice for neuroimaging data where multiple-testing across related spatial and temporal datapoints is a common problem [52], and where more conservative methods, like the Bonferroni correction, does not offer a good solution [53]. Results were deemed significant if the probability of type I error (false positives) was lower than 5%. On electrodes where the CNV showed significant group differences, the following procedure was adopted to reduce the possibility that artifacts were being studied. The post-stimulus interval was also entered into a point-by-point analysis and compared between groups applying the same statistical procedure described above. If the post-stimulus effects were non-significant (even if uncorrected), the pre-stimulus differences were accepted as genuine.

#### Time-frequency Analysis- Inter-Trial phase Coherence (ITC)

**Figure 2:**
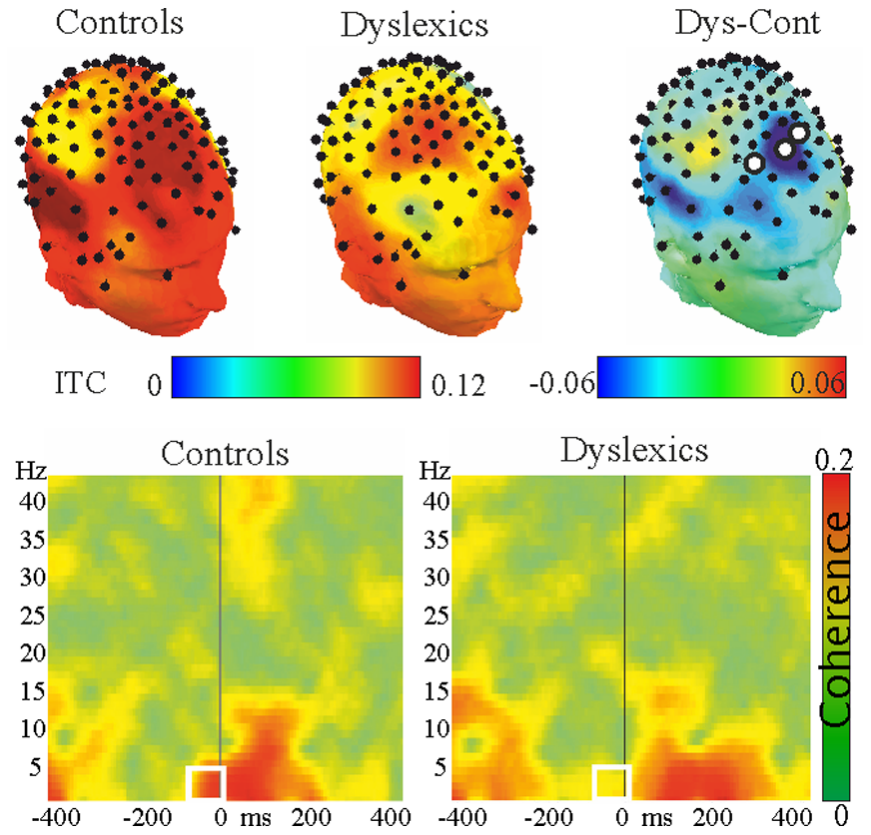
Time-frequency plots, 2 hz condition. The two lower panels show ITC across frequencies from 0 to 40(right panel) and controls (left panel). Dashed vertical lines indicate the time of stimulus arrival. Statistical analysis revealed significant group differences in the delta frequency range (∼0.5–4 Hz) in the prestimulus interval (−40–0 ms). Topographic headplots show ITC values over the whole head for both groups and the difference between the groups. Electrodes with significant group difference are marked with disks.

Time-frequency inter-trial coherence (ITC) analysis was applied to all trials using short-time Fourier transforms (STFT) with Hanning window tapering, resulting in a time-frequency landscape with a resolution of 4 ms in time and 0.98 Hz (from 0.9 to 40 Hz) in frequency. Oscillatory phase coherence was tested with 2-tailed point-by-point t-tests on the coherence values between the dyslexic and control groups, run across the three fronto-central electrodes (the electrodes are identical to those in the time-domain analysis; see Figure 2), and across the time points preceding stimulus presentation (−100 ms to 0 ms) in the delta frequency band (∼0.5–5 Hz). As in the time-domain analysis, p-values were corrected for the increased probability of false positives due to multiple-testing by using the FDR correction [52]. Results were deemed significant if the probability of type I error (false positives) was lower than 5%. The results demonstrated more coherence in controls than in dyslexics in a short time window before the stimulus (−40 ms –0 ms) in the delta (0.5–4 Hz) frequency range in the 2 Hz condition over the three fronto-central electrodes. This is shown in Figure 2. Statistics are reported in the results section.

Based on these coherence results, the prestimulus delta oscillation was thus selected for further analyses of instantaneous phase in both conditions. The trial-by-trial phase analysis also corrected for the potential artifacts caused by the STFT procedure, which can smear post-stimulus effects into the pre-stimulus time interval.

#### Band-pass filtering and trial-by-trial phase analysis

Prestimulus delta phase alignment was tested across all trials (target and non-target) on the electrode which showed the strongest ITC effect (FCz, see Figure 2). Prestimulus delta phase correlations with reaction time (RT) trial-by-trial were tested across all target trials, pooled across all subjects (see also [22], [23]), in order to examine the trial-by-trial pattern and the functional role that prestimulus delta phase plays in behaviour in developmental dyslexia.

For the extraction of instantaneous delta phase, the continuous (i.e. not epoched) data were entered into a 2^nd^ order causal (forward) Butterworth narrow band pass infinite impulse response (IIR) filter at 0.5–3 Hz. Although the causal (forward) Butterworth filter shifts phase, due to the narrow frequency band it does so in a systematic and linear manner, hence does not result in disturbances in the subsequent across-trials correlational analysis of phase. A causal (forward) filter was chosen because its impulse response runs only from ‘left to right’ along the time axis and not in both directions. Filters which do not shift phase are either applied as convolution (wavelet transform) or as two-directional filters, which are run both ways (forwards and backwards). The impulse responses wavelets and two-directional filters therefore run both ways along the time axis and consequently ‘smear’ around larger peaks. This ‘smearing’ can introduce artifacts into the prestimulus data, however that was not the case here, as illustrated by Figure S1. The impulse response of a relatively large ERP peak, for example related to visual perception or to motor response preparation, introduces distortions in the phase and amplitude of the oscillations in its neighbourhood (via convolution or via recursion running from right to left along the time axis). If the filter not only runs forwards along the time axis, as is the case with the causal (forward) Butterworth filter, but also backwards, then the impulse response of the large ERP peak might have a systematic distorting effect on prestimulus oscillations. This distortion in the phase and power of the prestimulus oscillation would be (incorrectly) correlated to the phase and amplitude of the large peak, resulting in probably significant, but false correlations between prestimulus phase and poststimulus evoked responses. With the causal filter used here, however, the possible distortion emanating from the large evoked potential would not have any effects backwards i.e. would not influence the phase pattern of the *prestimulus* interval. Note further that as each target was preceded by several non-target stimuli, large evoked potentials arising from the behavioral response to a previous target trial could not affect the next target trial’s prestimulus phase and RT correlations.

The filtered continuous data was entered into the Hilbert transform [54], [55] in order to extract instantaneous phase angles of the filtered frequency band (delta band). The phase angle is computed as the inverse tangent of the ratio of the real and imaginary parts of the complex-valued coefficients. The filtered and Hilbert-transformed continuous data was then epoched, and epochs previously marked as bad were rejected. The phase angle at the moment just preceding the presentation of the stimulus in each trial in each subject from all electrodes was subjected to further statistical analyses (i.e. −2 ms relative to the stimulus, as the sampling rate was 500 Hz). Phase analysis focused on data from the FCz electrode which showed the strongest (significant) ITC effects (Figure 2).

In order to assess the rhythmic entrainment of delta oscillations, phase locking trial-by-trial was tested by the Rayleigh test for the non-uniformity of circular data. Phase angle is circular and can take values within the circle, from –π to +π, or from 0° to 360°. If the distribution of phase angles at the moment just prior to stimulus presentation is not uniform, but has a certain preferred direction in its distribution for all participants, then the phase of the delta oscillation is entrained (i.e. not random). Hence a significant Rayleigh statistic suggests that the rhythmic excitability of neural assemblies is aligned with the rhythmic auditory stream [22], because the phase of neural oscillations is aligned predictively to the rhythm of the stimulation (in the present study Rayleigh statistics would confirm our findings on entrainment as measured by ITC). To test whether the mean phase was different between the two groups, mean phase values were also compared between the two groups with a circular multi-sample test for equal means. To see whether the spread of the distributions was different between the two groups the circular Kuiper test was used. A significant difference in the spread of the distributions would also indicate differences in signal-to-noise ratio between the groups. Further, to see whether delta oscillations were functioning as a mechanism for regulating response preparation in our rhythmic paradigm [23], we assessed whether RT could be predicted from the prestimulus delta phase angle by means of circular-linear correlations [22], [23], correlating prestimulus delta phase at the time point just before the stimulus occurred (−2 ms bin) and reaction time across all target trials in each group. If reaction time is correlated with the phase of the delta band oscillation just prior to the stimulus occurring, this would indicate that delta phase is functionally important for behaviour. For the circular statistics (non-uniformity, mean comparison, concentration and circular-linear correlations), the Matlab toolbox for circular statistics was used [57], [58]. Significance of the statistics was defined by 10,000 bootstrap iterations.

## Results

As shown in Table 1, our own test battery supported the dyslexia diagnosis for the experimental group. Although the experimental and control groups did not differ in verbal I.Q. (Wechsler Adult Scale of Intelligence, WASI) or nonverbal I.Q. (WASI), they differed significantly in their standard scores in reading (WRAT, *p* = .001) and spelling (WRAT, *p<*.001), phonological awareness (Spoonerism task, *p* = .044), digit span (*p* = .008), and RAN (*p* = .003). Further, sensitivity to syllable stress as measured by *d′* was significantly lower in the dyslexic group for both versions of the stress sensitivity task (Same Word, *p* = .013; Diff Words, *p*<.001).

**Figure 3:**
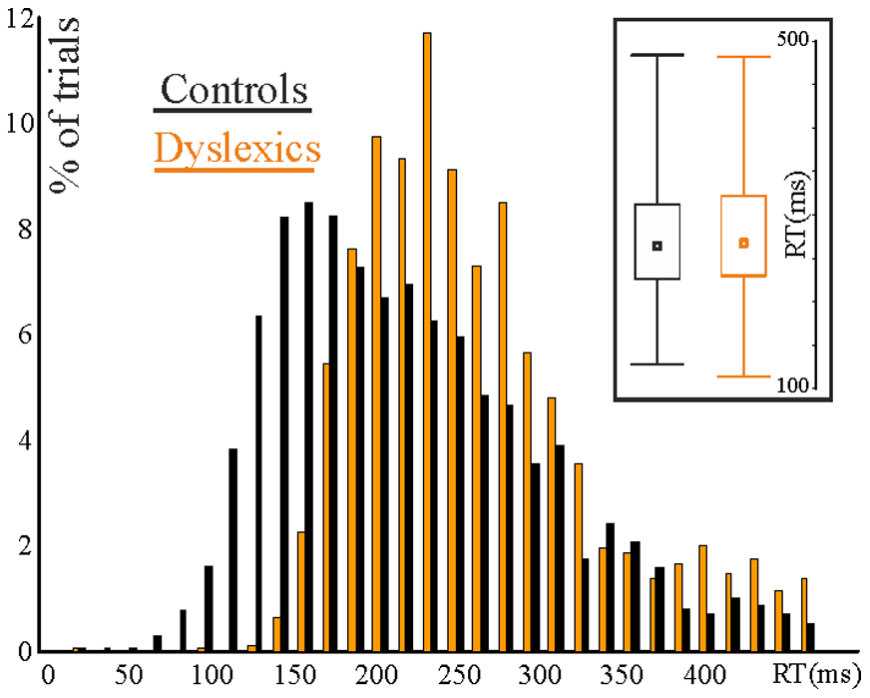
Distribution of Reaction Times (RTS) by group, 2 hz condition. Middle square indicates median RT; Box indicates the middle 75% of the distribution; Tails indicate min/max.

For the reaction time data we analysed 1890 responses (2 Hz) and 2126 responses (1.5 Hz) for the control group and 2084 (2 Hz) versus 2070 (1.5 Hz) responses for the dyslexic group. Mean and median reaction time to the white noise target was very similar between groups at 2 Hz and at 1.5 Hz (*controls* 2 Hz: mean 274 ms [s.d. 39.6], median: 270 ms [56.3], skewness: 0.69, kurtosis: 0.02; *dyslexic* 2 Hz mean 282 ms [s.d. 50], median: 268 ms [40.7], skewness: 0.87, kurtosis: 0.43; *controls* 1.5 Hz: mean 314 ms [s.d. 53.7], median: 304 ms [55.9], skewness: 1.18, kurtosis: 2.11); *dyslexic* 1.5 Hz: 301 ms [s.d. 44.5], median: 290 ms [45.1], skewness: 1.52, kurtosis: 3.97). Although there was a larger difference in mode, this also did not differ statistically between groups (2 Hz mode: *dyslexic*, 225 ms [56.2], *control*, 173 ms [59.1], adjusted *Z* = −1.05, *p*>.2; 1.5 Hz mode: *dyslexic*, 258 ms [47.2], *control*, 296 ms [59.2], adjusted *Z* = 0.26, *p*>.7). Both groups’ RT distribution differed significantly from the normal distribution, *p′*s = 0.01 for both. In addition, the RT distributions between groups were stochastically different in both conditions (2 Hz: adjusted Z = −1.985, p = 0.047; 1.5 Hz: adjusted Z = 3.4, p<0.0001). Thus, although overall the task was not more difficult for the dyslexics (RTs were similar), the different distributions of response time are suggestive of differences in underlying neural processing. The RT distribution in the 2 Hz condition is shown in Figure 3.

Topographic head plots and the ERPs by group showing the CNV for each condition were provided as Figure 1. Statistical analysis revealed significant group differences in CNV amplitude at the right fronto-central electrode site in both conditions beginning 30 ms before the arrival of the stimulus (all electrodes uncorrected, p<0.005; all electrodes corrected p<0.048, average F(1,30) = 4.32, Cohen’s d = 0.36). This ERP difference suggests differential preparatory brain activity between groups to the upcoming event in the rhythmic stimulus stream, even though tone presentation was rhythmically isochronous and therefore predictable. The smaller dyslexic CNV is suggestive of less neural preparation for the next event in the stream. Importantly, there were no significant group differences when the post-stimulus interval was analysed alone. This suggests that the significant pre-stimulus effects are not an artifactual consequence of post-stimulus effects affecting the magnitude of the CNV.

The results of the ITC analysis were shown in Figure 2. ITC in the delta frequency band showed significant differences between the two groups over the three fronto-central electrodes (all electrodes uncorrected p<0.005; all corrected p<0.043, Cohen’s d = 0.6).

**Figure 4:**
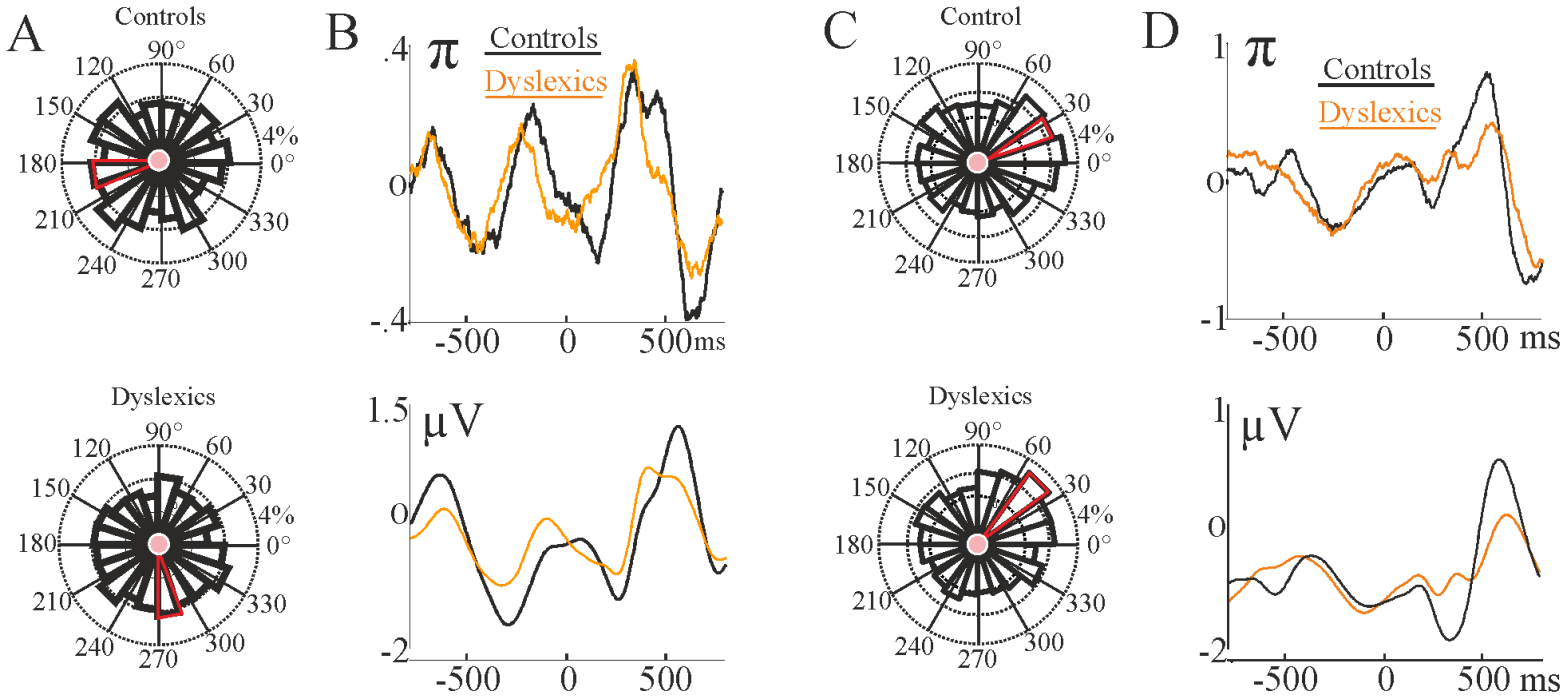
Phase entrainment of prestimulus delta oscillation by group. Panel A: Phase distribution in the 2 Hz condition is plotted as a rose diagram (from the electrode approximately corresponding to FCz in the 10–20 electrode location system, see figure 1), with the radial extent of the petals representing the probability of a given phase range. Top row depicts controls, bottom row depicts dyslexics. The red petal indicates the mean angle for each group. Panel B: Upper: Phase angle (depicted via π) of the delta oscillation along the whole epoch (from −800 to 800 ms) for both groups. Lower: Average EEG trace showing the delta oscillation along the whole epoch (from −800 to 800 ms) illustrating the ongoing nature of the delta oscillation for both groups. Panel C and D show analogous plots to Panel A and B, but for the 1.5 Hz condition.

The results of the inter-trial delta phase analyses are shown in Figure 4 for the 2 Hz condition (panels A and B) and the 1.5 Hz condition (panels C and D). In both conditions (2 Hz, 1.5 Hz), the data analysis indicated that the instantaneous phase angle of the delta oscillation (0.5–3 Hz) was significantly aligned across trials prior to the stimulus (−2 ms bin) in both groups. Thus the instantaneous delta phase values at the time of the next expected stimulus showed significant alignment for both groups in both conditions (Figure 4). The rose diagrams (Panel A for the 2 Hz condition and Panel C for the 1.5 Hz condition) depict phase entrainment in terms of the distribution within the circle of delta phase values, with the radial extent of the circle segments representing the probability of a given phase range. As can be seen from the petals marked in red, the average phase angle is different between groups, particularly at 2 Hz. The mean value of the preferred phase angle also changes between the 2 Hz and the 1.5 Hz conditions, for both groups. Nevertheless, the differences in the means (as tested by the circular multi-sample test for equal means) were not statistically significant for these adult groups. The phase angle of the delta oscillation along a whole epoch (measured from −800 ms before the target to +800 ms, depicted using π) is also shown (Figure 4, Panels B and D, upper trace, for the two conditions) to depict the oscillation. The average, narrow-band pass filtered delta EEG time-domain trace is additionally shown (Figure 4, Panels C and D, lower trace) to confirm the ongoing nature of the oscillation. Overall, the passive entrainment data show more similarities than differences between the groups, except for the strength of entrainment, as measured by ITC. As noted earlier, the strength of delta entrainment differed significantly between the two groups over right frontal and fronto-central electrode sites in a short time window before the stimulus (−40 ms –0 ms) for the 2 Hz rate, guiding the phase alignment analysis.

**Figure 5:**
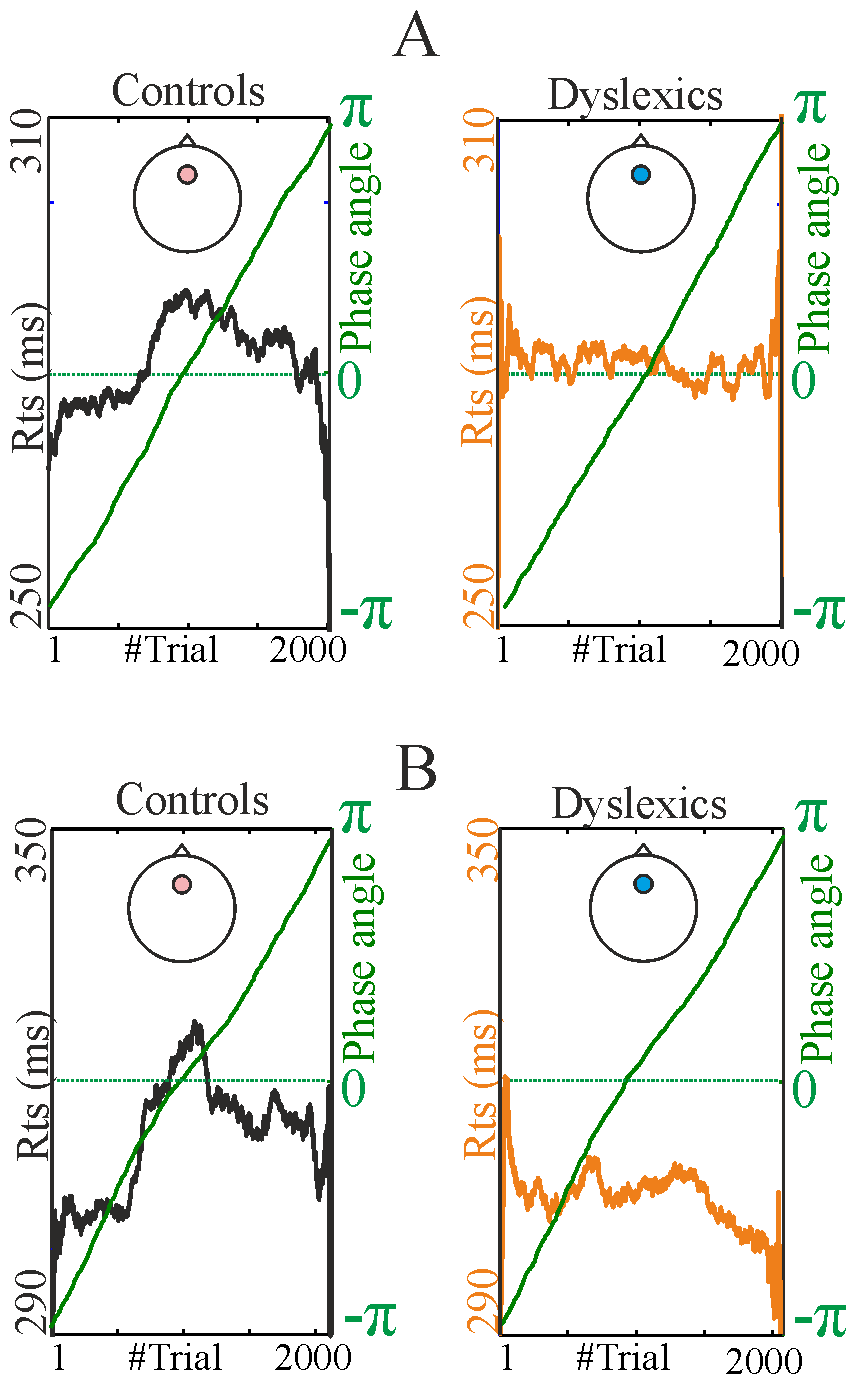
Task performance correlated with delta phase by conditions. Panel A: 2 Hz condition. Panel B: 1.5 Hz condition. Both panels show RT-phase correlations from the most representative electrode (from the electrode approximately corresponding to FCz in the 10–20 electrode location system, see Figure 1). Trials are sorted according to their phase angle in ascending order. Trials with a prestimulus delta phase of –π are followed by trials with prestimulus delta phase ascending towards +π. The phase angle across the trials is represented by the green line. The corresponding RT value is represented by the blue line. The non-linearity of the blue line with respect to the green line for the control participants indicates that prestimulus delta phase predicts RT. For visualisation purposes the RT plots are smoothed using a 500 point sliding window.

Furthermore, when we tested whether reaction time could be predicted from prestimulus delta phase angle in the target trials, we found that the phase of the delta oscillation just before the stimulus (−2 ms) predicted behavioural reaction time in the control group only. This is shown in Figure 5 (Panel A: 2 Hz condition, Panel B: 1.5 Hz condition). As in linear correlations, circular correlations can take a value between 0 and 1, with 0 corresponding to no relationship between phase angle and response time across trials, and 1 corresponding to a perfect correlation between phase angle and response time across trials. The figure demonstrates that the significant effects for the control participants were consistent in both the 2 Hz condition (circular *r* = 0.16, *p*<0.01) and in the 1.5 Hz condition (circular *r* = 0.13, *p*<0.05). However, prestimulus delta phase did not predict reaction time in the dyslexic group in either condition (2 Hz: *r* = 0.04, *p*>0.1; 1.5 Hz: *r* = 0.08; *p*>0.1). It is important to emphasize that the lack of significance for the dyslexics was not due to a difference in the spread of the distributions between the two groups (i.e. the dyslexic data were not noisier), as the circular Kuiper test did not show significant differences (*p*>.3). This suggests that prestimulus delta phase influenced behaviour for the control participants only. However, although the finding that prestimulus delta phase predicted RT in the control group is consistent with prior studies [23], the strength of the correlations did not differ significantly between groups according to the statistical comparison of the *r* values (2 Hz: Fisher’s Z = 0.3, 1.5 Hz: Fisher’s Z = 0.12; both p>0.3). Figure 5 also shows the RT-phase correlation for both groups, with trials sorted according to phase angle in ascending order. As can be seen, for the controls, faster reactions were observed when the target was delivered during the rising slope of the oscillation (nearer to −π), as found in other paradigms with neurotypical adults [23]. In contrast, there was no apparent relationship between response speed and the phase of the oscillation for the dyslexic participants. Delta oscillations do not appear to be functionally related to reaction speed for the dyslexics.

**Figure 6:**
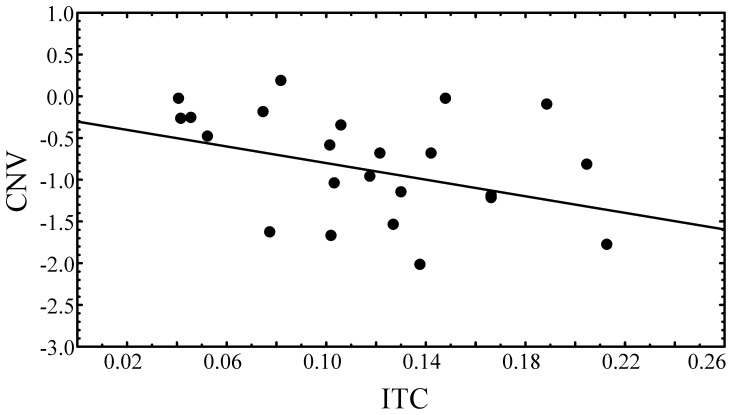
Correlation between ITC and CNV. CNV (in µV) values as a function of coherence (ITC) in the 2 Hz condition. The solid line indicates the regression line.

In order to investigate the expected relationship between low frequency oscillations and the CNV ERP component, average ITC values from the significant electrodes (as shown in Figure 2) and average CNV amplitude values from the electrodes showing the significant effect (Figure 1) were extracted for each participant. The correlation between the CNV and coherence at 2 Hz (ITC) was computed and was significant: *r* = −0.435, *p*<.04, see Figure 6. This relationship would be expected if the dynamics of low-frequency oscillations underlie the CNV in the time-domain [37], [38], [56]. The significant correlation further suggests that the pre-stimulus CNV observed here does not arise from artifacts emanating from the post-stimulus interval, since the pre-stimulus ITC is free from post-stimulus effects (as shown in Figure S1), and explains a significant portion of the variance of the CNV. Both pre-stimulus ITC and CNV theoretically reflect preparatory processes preceding the arrival of an expected stimulus (here, the next expected rhythmic beat). Hence both pre-stimulus ITC and CNV reflect hidden cognitive processes occuring in anticipation of the next sensory event, and are reduced in dyslexia.

When relations with the behavioural measures were explored (reading, spelling, sensitivity to syllable stress and phonological processing), both CNV amplitude and ITC strength at 2 Hz were related to better performance. Correlations are shown as Table 2. At the 1.5 Hz rate, fewer significant correlations were observed, nevertheless CNV amplitude was significantly related to most of the behavioural measures. The data suggest a relationship between the preparatory efficiency of the neural networks supporting rhythmic acoustic attention, and individual differences in phonological and reading development.

**Table 2:** Pearson correlations between ITC strength, CNV amplitude and the language measures for the two rhythmic presentation rates (2 Hz, 1.5 Hz).

	2 HzITC	2 HzCNV	1.5 HzITC	1.5 HzCNV
Reading standard score (WRAT)	0.50*	−0.45*	0.01	−0.45*
Spelling standard score (WRAT)	0.55**	−0.53**	0.04	−0.54**
Syllable Stress Same Word	0.54**	−0.45*	0.07	−0.54**
Syllable Stress Different Word	0.47*	−0.45*	−0.29	−0.53**
Spoonerisms	0.46*	−0.28	−0.05	−0.41°
RAN time in seconds	−0.45*	0.32	−0.09	0.46*
Digit span	0.39°	−0.37°	−0.13	−0.33

°p<0.1;

* p<0.05;

** p<0.01.

## Discussion

These data provide the first evidence for atypical functional neural rhythmic entrainment in developmental dyslexia. Although these well-compensated adult dyslexic participants did show modulation of synchronised delta oscillations by the rhythmic stimulus stream, the inter-trial coherence (ITC) of this modulation was significantly weaker than that of control participants at 2 Hz and the dyslexics also showed significantly less prestimulus contingent negative variation (CNV, a component implicated in predictive timing [38]), suggestive of less precise preparatory activity. Furthermore, both CNV amplitude and ITC strength significantly predicted sensitivity to syllable stress, phonological development and reading development, indicating a functional relationship between neuronal anticipatory entrainment in the delta frequency range and reading performance. In addition, when the relationship between oscillatory phase and rhythmic behaviour was examined, prestimulus instantaneous delta phase alignment was found to be unrelated to rhythmic auditory target detection in the dyslexics. For control participants, the instantaneous phase of the delta band oscillation played an important role in enhancing target detection, consistent with previous studies [23] (see also Figure 6). For dyslexics, the efficiency (reaction time) of the detection of individual auditory targets was unrelated to delta phase just prior to the occurrence of each of those targets. This could not be explained by reduced signal-to-noise ratio for the dyslexics or by less efficient overall responding (dyslexic mean, median and mode reaction times did not differ significantly from controls). However, the *r* value for the dyslexics (computed from pooled data) was not significantly different from that of controls, which could be due to the small sample size and large variability within the data. Therefore, the phase-RT results are inconclusive as to whether dyslexics differ from controls in their phase-behaviour relationship. Nonetheless, the significantly weaker oscillatory alignment in the delta band in dyslexics (lower ITC) and a significantly smaller CNV are both consistent with an impaired ability to create an efficient framework for auditory attention, despite the predictable rhythmic presentation. As would be expected on the basis of the temporal sampling theory [5], the oscillatory function of low frequency brain rhythms appears to be atypical in developmental dyslexia.

This disconnect between oscillatory delta phase and auditory attention in developmental dyslexia may imply that responding to task-relevant stimuli within a (slow) rhythmic temporal framework depends on different neural mechanisms in dyslexia. Anticipatory attending has been linked to oscillatory function in cognitive models, for example by Jones and her colleagues [35]. Weaker anticipatory processing by dyslexics was indicated here by both ERP and oscillatory findings.

It has been suggested that when operating in “rhythmic mode”, the brain uses low-frequency rhythms to provide a general oscillatory framework for attention [27]. Our data suggest that the dyslexic brain may not use low-frequency rhythms to govern attention in the same way as the non-dyslexic brain. The initial time-frequency inter-trial coherence analyses did not show oscillatory activity in the gamma band in response to the rhythmic stimulus streams, hence there is no evidence that delta oscillations in dyslexia are failing to reset gamma oscillations [58]. Rather, oscillatory entrainment was specific to the stimulation rate, as found in previous delta entrainment paradigms using rhythmic stimulation [22], [23]. However, a recent study utilising intracranial recordings in two patients with a very low frequency rhythmic stimulation rate (0.67 Hz) revealed amplitude peaks at both the stimulation rate and its harmonics within the delta band (i.e., 0.67 Hz, 1.33 Hz, 2 Hz, 2.67 Hz, and 3.33. Hz, see [24]). Phase alignment was found for the stimulation rate (0.67 Hz) and the second harmonic (1.33 Hz) only, with attention in both visual and auditory modalities related significantly to phase organization at these rates only.

Furthermore, consistent with temporal sampling theory [5], both delta ITC strength and CNV amplitude were significantly related to sensitivity to syllable stress, phonological development and reading development. Accurate encoding of the temporal structure of the speech signal (speech rhythm) is critical for phonological development, from infancy onwards [32], [59]. For phonological processing of speech, impaired rhythmic attending in the delta band would result in an impaired ability to predict when stressed syllables are likely to occur in the continuous acoustic stream (as the perception of P-centres would be impaired [34]). It would also affect the computation of the amplitude modulation phase hierarchies that underpin phonological structure across languages [7]. Goswami and Leong [7] pointed out that the child’s perception of global rhythm would depend on how efficiently the phase relations between low-frequency amplitude modulation patterns with different rise times were extracted, and that this would affect the development of well-specified phonological representations for words during language development. Impaired rhythmic attending would lead to phonological difficulties in the syllabic parsing and segmentation of the speech stream, and to reduced prosodic awareness and impairments in perceiving syllable stress, linguistic impairments that are indeed found in dyslexics and in poor readers [5], [12], [13], [14], [57], [60], [61]. If the perceptual effects of the *rise times* in the amplitude envelope and the *phase relations* between different temporal rates are processed inefficiently in dyslexia, then even a small difference in the function of delta oscillations in creating a rhythmic framework to support auditory attention would have important consequences for the development of the phonological lexicon in affected children. For example, atypical oscillatory function would result in differently-coded lexical representations, with prosodic information less well-represented. By this account, our finding that delta ITC strength and CNV amplitude differ in dyslexia could be relevant to the development of phonological processing [5], [7].

Amplitude fluctuations in the delta band in speech carry prosodic structure [7]. Neuronal oscillations in lower-frequency bands like delta would be critical for supplying the temporal *context* for processing a complex stimulus like the speech signal [8]. The lower frequency oscillations would act to direct more detailed processing of spectral content (e.g. spiking activity) to particular points in time. According to the amplitude modulation phase hierarchy perspective [7], [62], atypical rhythmic entrainment at 2 Hz would be expected to affect the ability to predict when stressed syllables are likely to occur in the continuous acoustic stream, and therefore would affect the representation of the temporal structure of spoken words – phonological representation. Atypical neuronal entrainment to the low frequency temporal information supporting prosodic structure would have effects throughout the phonological system, thereby affecting phonetic-level representations as well. Momentary acoustic input is not sufficient for decoding speech, as speech is not decoded on a linear basis in terms of rapidly successive acoustic events [63]. Rather, the temporal envelope of speech provides the longer temporal context within which the position of momentary changes in fine structure (spectral content) can be interpreted. The intrinsically hierarchical temporal nature of speech processing, captured by the amplitude envelope phase hierarchy, makes it plausible to propose that atypical cortical entrainment of oscillations below 10 Hz in dyslexia would have system-wide effects. Atypical delta function would affect how the dyslexic brain directs more detailed spectral processing to particular moments in time, perhaps leading to over-sampling at the gamma rate [42], [45].

In summary, our phase alignment data taken together with the significant group differences found for inter-trial coherence and contingent negative variation suggest that individuals with dyslexia do not use anticipatory mechanisms efficiently within the delta oscillatory stream to affect behaviour. The demonstration also has wider implications for the understanding of auditory attention and rhythmic behaviour [64], [65], [66]. Cognitive ‘dynamic attending’ theory [35] argues that beat regularity enables anticipatory attending. The current data suggest that beat regularity is not used by the dyslexic brain to anticipate auditory events in the same way as by the non-dyslexic brain. However, our participants were highly-compensated dyslexics attending university, and so data from children are required to study the efficiency of entrainment earlier in the developmental trajectory [46], [67]. Furthermore, the current data do not reveal the nature of the compensatory mechanisms that enabled equally efficient behavioural responding in the button press task by the dyslexics who participated in this study.

## Supporting Information

Figure S1 **Impulse response of digital filters.** Black line: simulated data of an 1600 ms long signal (sampling rate of 500 Hz) with zero values except for an impulse of one unit in the middle of the epoch. Red line: Impulse response of a zero-phase shift FIR filter (filter properties: 0.5–3 Hz bandpass, 128 points). As seen in the figure, the filter creates non-zero values of certain phase in the ‘pre-stimulus interval’. Blue line: Impulse response of the causal forward filter (filter properties: 3^rd^ order, 0.5–3 Hz bandpass). This filter does not create non-zero values in the pre-stimulus interval, hence does not result in pre-stimulus artifacts emanating from the evoked (post-stimulus) response.
(TIF)
