# Supplementary figures and images for: Differential Entrainment of Neuroelectric Delta Oscillations in Developmental Dyslexia
Source: PLoS One. 2013 Oct 18;8(10):e76608. doi: 10.1371/journal.pone.0076608 (PMC3799758; doi:10.1371/journal.pone.0076608)

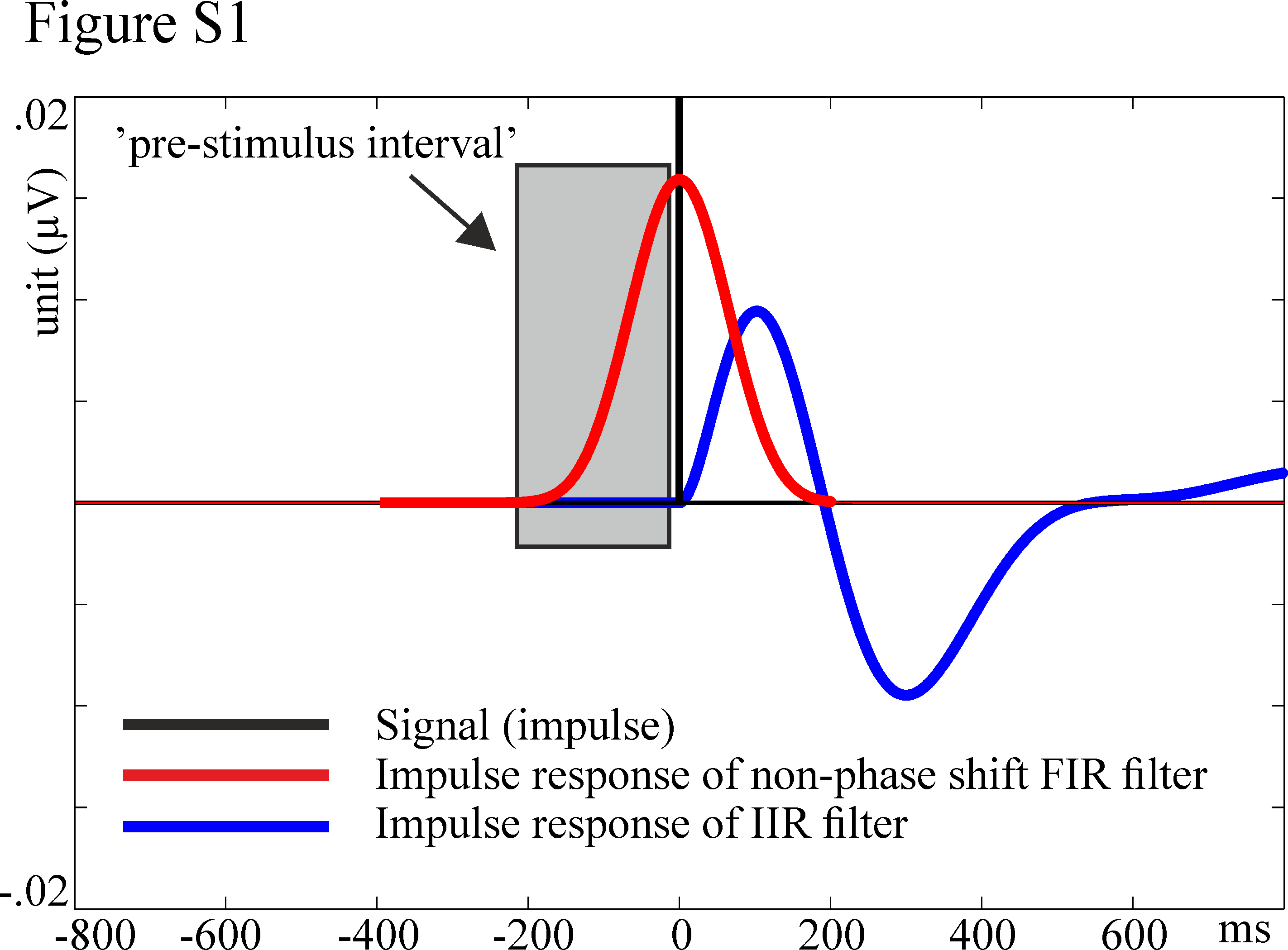

Supplement: Figure S1 — Impulse response of digital filters. Black line: simulated data of an 1600 ms long signal (sampling rate of 500 Hz) with zero values except for an impulse of one unit in the middle of the epoch. Red line: Impulse response of a zero-phase shift FIR filter (filter properties: 0.5–3 Hz bandpass, 128 points). As seen in the figure, the filter creates non-zero values of certain phase in the ‘pre-stimulus interval’. Blue line: Impulse response of the causal forward filter (filter properties: 3rd order, 0.5–3 Hz bandpass). This filter does not create non-zero values in the pre-stimulus interval, hence does not result in pre-stimulus artifacts emanating from the evoked (post-stimulus) response. (TIF) [file pone.0076608.s001.tif]
